# Items

**Published:** 1907-06

**Authors:** 


### ITEMS.

The State Civil Service Commission will hold examinations June 15, 1907, for the following positions: Court attendant, Erie county service, $1,200; custodian or messenger, Kings county offices, $720 to $1,000; elevator conductor, city and county hall, Buffalo, $540; police constable, $900, and watchman, $1.75 a day at the Niagara Reservation; resident physician, State Hospital for Crippled and Deformed Children, $900 and maintenance; superintendent of inspection, state board of charities, $2,500; veterinarian, health and agriculture departments, $7 a day: woman officer, state charitable institutions. $300 to $360 and maintenance.

The last day for filing applications for these positions is June 8. Full information and application forms may be obtained by addressing Charles S. Fowler, Chief Examiner, Albany.

---

THE OD CHEMICAL COMPANY announces its removal from 15 Cedar street to its new and commodious building, 61 Barrow street, New York.

---

MOET AND CHANDON White Seal 1900 Champagne, is the most palatable and efficient sparkling wine for use in case of sickness of which we have any knowledge. It is free from the objectionable features that often follow the use of some champagnes and is a positive aid to treatment when rapid stimulation is required, and in cases of nausea or obstinate vomiting. The JOURNAL is making this statement from knowledge obtained through its therapeutic employment in the sickroom, and from general clinical observation of the results of such application of this most delightful wine. A trial will satisfy the doubter. Pint bottles can be tapped with the champagne tapping device, or the patent rubber stopper may be employed, thus avoiding extravagant use or waste of the wine.

---

APPOLLINARIS, the "queen of table waters," is the most satisfactory of all sparkling waters in the sickroom. It is a refreshing beverage as well as a therapeutic agent of value; it agrees with a sensitive palate and it soothes an irritable stomach. The general use of Apollinaris, of course, is well understood; we are now discussing its merits from our experience with it in the sickroom. If physicians would prescribe it more frequently, it would be more gratifying to their patients and more satisfactory to themselves.

---

"Boss," shouted the big cook from the kitchen, "we have a lot of scraps out here that ain't working."

"Lot of scraps, eh?" replied the proprietor of the Shovedown lunchroom. "Well, mix them all together, add a little fiery tobasco sauce and then put a sign outside, 'Central America Pudding Today.' "—Chicago News.

